# Decreased Expression of the High Mobility Group Box 1 (*HMGB1*) Gene in Peripheral Blood in Patients with Mild or Moderate *Clostridioides difficile* Infection

**DOI:** 10.3390/microorganisms8081217

**Published:** 2020-08-11

**Authors:** Jacek Czepiel, Grażyna Biesiada, Ewelina Pitera, Paweł P. Wołkow, Mateusz Michalak, Aleksander Garlicki

**Affiliations:** 1 Department of Infectious and Tropical Diseases, Jagiellonian University Medical College, 30-688 Krakow, Poland; gbiesiada@op.pl (G.B.); agarlicki@gmail.com (A.G.); 2 Center for Medical Genomics OMICRON, Jagiellonian University Medical College, 31-034 Krakow, Poland; ewelina.pitera@uj.edu.pl (E.P.); pawel.wolkow@uj.edu.pl (P.P.W.); 3 Department of Pharmacology, Jagiellonian University Medical College, 31-531 Krakow, Poland; 4 Ludwik Rydygier Hospital in Krakow, 31-826 Krakow, Poland; michalakcontact@gmail.com

**Keywords:** genetic, infection, inflammation, pathogenesis

## Abstract

Cytokines are mediators of inflammation induced in the course of *Clostridioides difficile* infection (CDI). High Mobility Group Box 1 (HMGB1) is a cytokine playing an important role in the pathogenesis of numerous inflammatory and autoimmune diseases. The aim of the study was to assess the *HMGB1* gene expression in the course of CDI. We have performed a prospective case-control study- including 55 adult patients, among them 27 with CDI, who were hospitalized from October 2018 to February 2020 and 28 healthy volunteers. We assessed: a complete blood count with differential leukocyte count, blood creatinine, albumin, and C-reactive protein (CRP) levels. Then, the expression of the *HMGB1* gene was evaluated using quantitative Real-Time PCR. Patients with CDI were found to have a significant increase in white blood cells (WBC), neutrophil count, and CRP levels, they also exhibited decreased levels of albumin compared with controls. The *HMGB1* gene expression was significantly lower among patients with CDI compared with the control group and significantly, inversely correlated with CRP level in blood. In conclusion, we have observed a decreased expression of the *HMGB1* gene in peripheral blood of patients with mild or moderate CDI, which hypothetically could reflect their diminished capability to fight the pathogen.

## 1. Introduction

*Clostridioides difficile* infection (CDI) is caused by anaerobic bacilli of the species *Clostridioides difficile* (*C. difficile*), formerly known as *Clostridium difficile,* widespread in the human environment [1]. In the last two decades, the incidence and severity of CDI have been growing worldwide, reinforcing it as one of the most eminent nosocomial infections of modern healthcare. Infection occurs through the fecal-oral route, the most important risk factors for the development of the disease are the use of antibiotics, hospitalization, and older age. The pathogen itself is not directly invasive- its virulence factors are enzymes it produces, such as collagenase, hyaluronidase, chondroitin sulfatase, and toxins, which destroy the intestinal epithelium cytoskeleton and stimulate adhesion of neutrophils to the intestinal wall, causing inflammation and loss of both the integrity and functionality of the intestinal mucosa. Cytokines: interleukin 8 (IL-8), interleukin 1β (IL-1β), interleukin 6 (IL-6), tumor necrosis factor α (TNFα), interferon γ (IFN γ), and leukotriene B4 also play an important part in CDI pathogenesis [2,3]. The clinical picture of CDI is diverse and ranges from asymptomatic carrier status, through various degrees of diarrhea, to most severe colitis often leading to death. The prevalence of *C. difficile* colonization during hospitalization correlates to the length of stay, and in a few weeks can reach even 50% of patients [3,4,5,6]. However only some become symptomatic [7], the reason for this remains unknown. Moreover, the severity of CDI may vary even between people similar in age and co-morbidities [8]. Genetic diversity, reflected in differences in immune system functionality of the patients may, at least partially, be a factor in CDI risk and/or variance of its course.

High Mobility Group Box 1 (HMGB1) is a cytokine located in the cell nucleus. There, it plays a vital part in maintaining chromosome structure and DNA damage prevention [9]. During the cellular stress response, it is released into the cytosol, and subsequently extracellularly, where it mediates the inflammatory, defense response and other cellular processes [9,10,11]. Efron et al. assessed genetic profiles of leukocytes in trauma patients, demonstrating an increased expression of the *HMGB1* gene in the subgroup of patients who developed CDI [12].

Therefore, the aim of our study was to prospectively assess *HMGB1* gene expression in the course of CDI.

## 2. Materials and Methods

### 2.1. Study Population

We prospectively studied 55 patients. The study group included 27 patients with mild or moderate CDI hospitalized at the Department of Infectious and Tropical Diseases, University Hospital in Krakow, Poland, from October 2018 to February 2020, and 28 healthy volunteers. All individuals were of Caucasian race. Infection was confirmed by detection of *C. difficile* antigen and toxins in feces using the TOX A/B Wampole Quick Check Complete test kit (TechLab Inc., Blacksburg, VA, USA). When the test came back positive for the antigen but negative for the toxin, the test for the *C. difficile* toxin was repeated with the *C. difficile* Toxin ELISA A/B II test kit (TechLab Inc., Blacksburg, VA, USA). A CDI case was defined as a patient with symptoms of CDI and positive laboratory test(s) according to the *European Society of Clinical Microbiology and Infectious Diseases* (ESCMID) guidelines [13]. Exclusion criteria included the presence of any CDI episode in control group, and the presence of acute or chronic inflammatory diseases in both groups. Patients with any infection in the control group, and any other infection than CDI in the CDI group were also excluded. All subjects had given their informed consent for inclusion before they participated in the study. The study was conducted in accordance with the Declaration of Helsinki (as revised in Brazil 2013). The study protocol was approved by the Ethics Committee of Jagiellonian University, ID number 1072.6120.211.2017.

### 2.2. Blood Tests

Blood samples were collected in the morning, between 7.00 and 8.00 a.m. after an overnight fast. They were taken at the beginning of infection, no later than 48 h after CDI diagnosis. We assessed the following: complete blood count with differential leukocyte count, serum creatinine, albumin, and C-reactive protein (CRP) levels. All tests were performed according to generally accepted standard procedures.

### 2.3. Gene Expression Assay

Whole blood was collected into 5 mL EDTA-treated tubes. Buffy coat fractions were prepared by centrifugation of the collection tubes at 1500× *g* for 15 min at room temperature. Next, the buffy coats were mixed in equal volume of DNA/RNA Shield (2× concentrate) (Zymo Research, Irvine, CA, USA) and samples were stored in −80 °C. RNA was isolated from 500 µL of a sample by using the Quick-RNA™ Miniprep Plus Kit (Zymo Research, Irvine, CA, USA), following the manufacturer’s instructions. One hundred and eighty nanograms of extracted RNA per sample were reverse-transcribed to cDNA by a High Capacity RNA-to-cDNA Kit (Applied Biosystems, Waltham, MA, USA). Quantitative RT–PCR was performed in triplicate with 2 μL of cDNA, 1× TaqMan^®^ Fast Advanced Master Mix and 1× TaqMan^®^ Gene Expression assay *HMGB1* (ID: Hs01923466_g1), glyceraldehyde-3-phosphate dehydrogenase *GAPDH* (ID: Hs02786624_g1), and actin beta *ACTB* (ID: Hs01060665_g1) (Applied Biosystems, Foster City, CA, USA) in a final reaction volume of 20 μL. Samples were amplified by the CFX96 Touch Real-Time PCR System (Bio-Rad, Pleasanton CA, USA) under the following thermal profile: initial incubation at 95 °C for 2 min, 40 cycles of denaturation at 95 °C for 1 s followed by annealing and extension at 60 °C for 20 s. Mean quantitation cycle (Cq) values of *HMGB1* were normalized to the geometric mean of *GAPDH* and *ACTB*, which were selected as stable, housekeeping genes [14,15,16]. Data were expressed for each sample as ΔCq, which is the difference between the Cq *HMGB1* value and the geometric mean of Cq values of *GAPDH* and *ACTB* for a particular sample.

### 2.4. Statistical Analysis

All data are presented as medians with lower (Q_25_), and upper (Q_75_) quartiles. Normal distribution of variables was analyzed using the Shapiro-Wilk test. Differences between study groups were determined using the Mann-Whitney test if normality was not observed. Correlation between selected variables was evaluated using Spearman’s rank correlation. Calculations were performed using StatSoft, Inc. (2011), STATISTICA, version 13.3 statistical software licensed for Jagiellonian University, and statistical significance was defined as *p* ≤ 0.05.

## 3. Results

The study group included 27 patients with mild or moderate CDI hospitalized at the Department of Infectious and Tropical Diseases, University Hospital, in Krakow, median age of 71 years, and 28 healthy volunteers median age of 69 years. Patients with CDI were found to have a significant increase in white blood cells (WBC), neutrophil count, and CRP levels, they also exhibited decreased levels of albumin compared with controls. *HMGB1* gene expression was significantly lower in the CDI group compared with the control group (Table 1).

**Figure 1:**
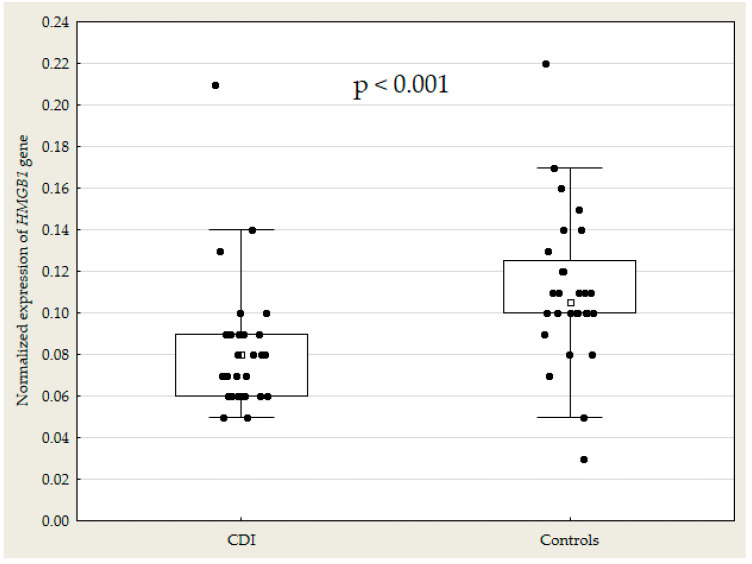
Normalized expression of *HMGB1* gene in CDI (*n* = 27) and control (*n* = 28) groups. Box represents 25th and 75th percentile of values with median values marked within the boxes. Whiskers represent 5th–95th percentile. CDI, *Clostridioides difficile* infection; *HMGB1*, high-mobility group box 1.

Graphical representation of the data on *HMGB1* gene expression is provided in Figure 1.

Next, a possible correlation between *HMGB1* gene expression and the selected blood parameters was assessed. In the control group, as predicted, no significant correlation was found, whereas in the CDI group there was a statistically significant inverse relationship between *HMGB1* gene expression and CRP level. No other significant relationships were found between the remaining parameters (Table 2). Research data are available as supplementary material.

## 4. Discussion

### 4.1. The Impact of Genetic Factors on the Course of Clostridioides Difficile Infection

Studies assessing the impact of genetic factors on the course of CDI have, so far, been very few. Garey et al. demonstrated the correlation of region 251 of the IL-8 gene polymorphism with higher recurrence of CDI, and an increase in fecal IL-8 concentration [17]. Jiang et al. have shown in two studies that an AA genotype in the 251 position of IL8 is an important predictor of primary CDI [18,19]. In our previous study, we have shown that the presence of IL-8 +781 T/C polymorphism is associated with the severity of CDI [20]. IL-8 is key in CDI pathogenesis, promoting chemotaxis of neutrophils to an infection site, which in turn initiates inflammation of the intestinal mucosa and emergence of symptoms [2,21]. The interleukin-4 associated single nucleotide polymorphism (SNP) rs2243250 has been associated with development of CDI in Inflammatory Bowel Disease (IBD) patients [22]. Jose et al. showed that homozygosity for leptin receptor Q223R SNP significantly increases the risk of peak peripheral WBC count >20 × 10^9^/L, which is an indicator of adverse outcomes. They have also demonstrated in a murine model of CDI, that mice homozygous for the same SNP exhibit a higher leukocyte count in blood and tissue, exaggerated tissue inflammation and higher mortality, compared with control mice [23].

### 4.2. The Role of HMGB1 in Clostridioides Difficile Infection

HMGB1 plays an important role in the inflammatory process. It stimulates cell migration to an inflammation/injury site, which has been demonstrated on neural cells, smooth muscle cells, tumor cells, monocytes, and neutrophils [24,25,26,27,28]. It also aids in identification of bacterial products, boosting the innate immune response [29]. Through binding to cellular receptors, such as toll-like receptor 4 (TLR4), TLR9, and RAGE, HMGB1 activates innate immune cells, endothelial cells to produce proinflammatory cytokines, chemokines, tissue factor or adhesion molecules [10,29,30,31,32,33]. Furthermore, HMGB1 inhibits elimination through phagocytosis of apoptotic neutrophils, impeding resolution of inflammation [29]. So far, the role of HMGB1 has been established in inflammatory processes of conditions, such as arthritis, hepatitis, sepsis, rheumatoid arthritis, and systemic lupus *erythematosus* [34,35,36], as well as the pathogenesis of atherosclerosis and cancer [37,38].

However, studies examining the potential role of HMGB1 in CDI pathogenesis are lacking. It has been shown in vitro that HMGB1 may be involved in acute inflammation induced by the *C. difficile* toxin A. Liu et al. in their study demonstrated that HMGB1 is released from the nucleus into the cytoplasm and subsequently, extracellularly in response to *C. difficile* toxin A (TcdA). Pre-treatment with glycyrrhizin, an HMGB1 inhibitor, delays TcdA-induced cell rounding. Moreover, they have shown that inducing exogenous rHMGB1 increased MPO activity and intestinal injury in the ‘ileal loop’ surgical model. A statistically significant increase in mRNA expression of several proinflammatory cytokine genes (TNF-α, IL-1β, IL-6, keratinocyte chemoattractant-KC) in comparison to phosphate buffered saline (PBS) stimulation has been demonstrated. Interestingly, the effect observed after introducing rHMGB1 was similar to that of TcdA [39]. In another study the HMGB1 protein was involved in endoplasmic reticulum stress induced by the TcdA [9]. Gu et al. have shown that treatment with recombinant *C. difficile* toxin B (rTcdB) promotes the release of HMGB1 from CT26 cells in a time-dependent manner. The HMGB1 knockdown CT26 cells treated with rTcdB reduced their ability to produce IFN-γ, a cytokine important in CDI pathogenesis [40]. Chumbler et al. have shown that TcdB induces HMGB1 release from the HeLa cell line [41].

### 4.3. HMGB1 Gene Expression in Different Infections

Apart from the aforementioned study of Efron et al. [12], *HMGB1* expression in CDI patients has been an underexplored subject. Studies delving into the changes in that expression in the course of different infections have, similarly, been few and far between. There has been an observed reduced expression of the *HMGB1* gene in chronic *Hepatitis B virus* (HBV) infected patients, which hypothetically could lead to impaired DNA damage repair and favor development of hepatocellular carcinoma [42]. In contrast to that, an increased expression of the HGMB1 gene was observed in *Hepatitis E virus* (HEV) infected patients [15]. In bacterial infections, HMBG1 gene expression was assessed in active pulmonary tuberculosis, where it was increased in comparison to asymptomatic individuals tested for latent tuberculosis [43]. In an animal model of sepsis, expression levels of *HMGB1* mRNA in the liver, lungs, kidneys, and small intestine of rats were markedly increased [44]. In another study, *Herpes simplex type 2 virus* (HSV-2) infection reduced *HMGB1* expression in HEC-1 cells, derived from human endometrial cancer, which were used as a model of epithelial cells [45].

As mentioned above, genetic diversity may at least partially explain why the severity of symptoms varies so drastically in people similar in age and co-morbidities. In our study, the participants with CDI exhibited clear signs of infection, substantiated by the considerably increased values of WBC and CRP. *HMGB1* is an important factor in the cellular defense response. It could be theorized that the observed decrease in expression of *HMGB1* in CDI patients is a reflection of the system’s diminished capability to fight the pathogen. That hypothesis is supported by the significant inverse correlation between *HMGB1* expression and CRP demonstrated in our study. The lower the expression of *HMGB1*, the higher CRP was found in the blood. However, it remains unclear why the decrease in *HMGB1* expression occurs, and whether it is a primary defect making that particular group of people vulnerable to developing symptoms of CDI or a secondary reaction based on a yet unexplained mechanism in the course of CDI. Further research into the subject is needed. In our study, we assessed the *HMGB1* gene expression in the early phase of infection, which would further support the hypothesis of genetic predisposition towards CDI. Leaning on in vitro research, the increase in *HMGB1* expression in the course of different infections was fast. In a study by Zhang et al. in an animal model of sepsis, this increase was detectable in 12 h [44]. Bearing that in mind, should the increase in *HMGB1* gene expression stem from the infection alone, it would be pronounced in our patients, as well. No such change in our study suggests, again, that it could be a primary defect making that particular group of people vulnerable to developing symptoms of CDI. Decreased expression of *HMGB1* has also been observed in the course of HBV infection. It is worth noting that no significant changes were observed comparing acute HBV patients with healthy controls. Generally, only a minority of patients develop chronic hepatitis B, and, in the study by Mukherjee et al., only in this group was a decrease of *HMGB1* gene expression observed. This observation supports the notion that a functioning *HMGB1* gene may be an important element in combating HBV [42].

Fecal HMGB1 was found to be a marker of intestinal inflammation, as it was not only significantly increased in the stool of Inflammatory Bowel Disease (IBD) patients, compared with controls, but also indicative of the course of the disease. Moreover, HMGB1 was a very sensitive marker of persistent local gut inflammation in IBD patients with clinically quiescent disease [46,47]. In our study, we did not include patients with severe or fulminant CDI, but even in patients with mild or moderate CDI, a significant increase in CRP was observed, possibly indicating a systemic inflammatory response in addition to the local inflammation in the colon. Although we did not explore a possible association between the expression of *HMGB1* and polymorphisms in this gene locus in our patients, such a relationship was theorized by many researchers. Kornblit et al. postulated this relationship based on in silico modeling, while others demonstrated it in pneumonia and cancer [48,49,50]. Several polymorphisms in gene loci of microRNAs regulating *HMGB1* expression were also identified [51].

The main limitation of our study was that we did not include patients with severe CDI. Secondly, we measured the *HMGB1* gene expression only at a single time point of infection. Moreover, we cannot exclude the possibility that CRP level is not a comprehensive marker of systemic inflammation in our patients due to potential co-morbidities with an inflammatory component, such as autoimmune diseases, pericarditis, tissue injuries, and other relatively commonly observed conditions. This may require careful exclusion of such co-morbidities in future study cohorts or inclusion of only those patients who are at the stage of remission while being recruited for a potential study of a similar scope.

## 5. Conclusions

We observed a decreased expression of the *HMGB1* gene in peripheral blood of patients with mild or moderate CDI, which hypothetically could reflect their diminished capability to fight the pathogen. Future investigations should also include patients with severe CDI. It would be beneficial to assess whether the expression of *HMGB1* changes at other time points of infection, especially at the onset and toward the end of infection.

## Figures and Tables

**Table 1:** Results of the assessed parameters in the study groups.

Parameter	CDI Group		Control Group		*P*
	*n*	Median (Q_25_–Q_75_)	*n*	Median (Q_25_–Q_75_)	
Age (years)	27	71 (67–84)	28	69 (65–72)	0.17
WBC (×10^3^/µL)	27	8.9 (6.8–12.3)	28	5.8 (5.1–6.8)	<0.001
neutrophils (×10^3^/µL)	26	6.5 (4.4–9.7)	28	2.9 (2.4–3.9)	<0.001
creatinine (µmol/L)	27	82 (58–121)	28	76 (69–90)	0.49
albumin (g/L)	25	28.6 (24.1–33)	20	40.3 (34.9–41.9)	<0.001
CRP (mg/L)	27	74 (18–120)	26	2.4 (1–3.8)	<0.001
ΔCq	27	3.71 (3.44–4.09)	28	3.25 (3.02–3.35)	<0.001
Normalized Expression of *HMGB1* gene	27	0.08 (0.06–0.09)	28	0.11 (0.10–0.13)	<0.001

CDI, *Clostridioides* *difficile* infection; CRP, C-reactive protein; *HMGB1*, high-mobility group box 1; *n*, number of patients; Q_25_, lower quartile; Q_75_, upper quartile; WBC, white blood cells; ΔCq, difference between the quantification cycle of the analyzed gene and reference genes. *P*: *p* value.

**Table 2:** Correlations between *HMGB1* gene expression and assessed blood parameters.

Parameter Correlated with *HGMB1* Gene Expression	CDI Group		Control Group	
**Parameters compared**	r	*p*	r	*p*
WBC	−0.29	0.14	−0.16	0.41
Neutrophils	−0.18	0.39	−0.28	0.16
Creatinine	−0.02	0.91	−0.13	0.50
Albumin	0.40	0.07	0.42	0.07
CRP	−0.42	0.03	−0.09	0.67

CDI, *Clostridioides difficile* infection; CRP, C-reactive protein; *HMGB1*, high-mobility group box 1; *p*, *p* value; r, correlation coefficient, WBC, white blood cells.

## Supplementary Materials

The following are available online at https://www.mdpi.com/2076-2607/8/8/1217/s1, Research data.
